# Microbial neuroscience: The gut microbiota as a cognitive layer

**DOI:** 10.1016/j.treopn.2026.06.006

**Published:** 2026-08

**Authors:** Philip W.J. Burnet

**Affiliations:** 1 Department of Psychiatry, University of Oxford, Oxford OX3 7JX, UK

**Keywords:** distributed cognition, microbiome–gut–brain axis, information processing

## Abstract

Cognition is traditionally viewed as a brain-centred process, with gut microbes treated primarily as modulators of physiology and behaviour. Recent advances in microbiome research suggest that microbial communities can exhibit history-dependent functional states, adapt their outputs, and dynamically interact with neural systems. This opinion article proposes a distributed model of cognition in which the gut microbiota acts as an intermediate information-processing layer that may influence cognitive states through memory-like persistence and closed-loop feedback with the brain. By integrating concepts from systems neuroscience, microbiology, and cognitive theory, this framework reframes the microbiota as a potential functional contributor to cognition and outlines experimental approaches for testing causal microbiota–gut–brain interactions in behaviour and decision-making.

## Significance

Cognition is traditionally seen as confined to the brain, yet the gut microbiota forms a semiautonomous layer that encodes memory-like states and influences neural circuits. Through bidirectional feedback with the brain, microbes shape decision-making, emotional regulation, and adaptive behaviour, extending the boundaries of distributed cognition. Integrating closed-loop experiments, computational models, and multi-scale neurobiological approaches, this perspective reframes the microbiota as a functional contributor to cognition, with broad implications for neuroscience, cognitive science, and translational research.

## Rethinking cognition: The microbiota as a cognitive partner

The **microbiota–gut–brain axis (MGBA**, see Glossary) is traditionally framed as a bidirectional communication network: microbial communities, metabolites, and immune signals influence brain function, while the brain regulates gut activity, which in turn shapes microbial composition and community structure. Although this circuit-like perspective is well-established, it implicitly treats cognition as a brain-centred phenomenon, casting microbes merely as modulators. In this study, ‘modulation’ refers to the directional influence of microbial or host-derived signals on physiological or neural processes, whereas system-level coupling refers to bidirectional interactions across microbial and host systems that collectively contribute to cognitive states. Emerging evidence invites a broader view, positioning the MGBA as a distributed cognitive system in which microbial ecosystems participate in memory-like (i.e., history-dependent) functional encoding, information processing, and behavioural modulation. Integrating insights from systems neuroscience, microbiome research, and theories of extended cognition, it is proposed that microbes may be conceptualised as semiautonomous functional contributors within an information-processing network, shaping cognition through history-dependent functional processes and real-time feedback.

Building on this perspective, this opinion article proposes a distributed cognition framework for the microbiota–gut–brain axis in which cognition emerges from interactions across three coupled systems: (i) neural processes in the brain, (ii) physiological and immune processes in the body, and (iii) adaptive, memory-like processes within microbial communities. In this framework, microbial activity may contribute to cognition through three core functions: (i) encoding experience in persistent, dynamic states, (ii) modulating host signalling via diverse functional outputs, and (iii) participating in continuous physiological and behavioural feedback loops with neural systems, in which microbial activity influences host signalling and behaviour, while host neural, endocrine, immune, and behavioural states continuously reshape microbial composition and function. Together, these processes position the microbiota as a functional component of cognition rather than a peripheral modulator.

In this context, ‘distributed cognition’ refers to information processing that spans neural and nonneural biological systems; ‘memory-like processes’ describe persistent microbial states shaped by prior host–environment interactions, and ‘closed-loop feedback’ denotes continuous, bidirectional coupling between microbial activity and brain function. Importantly, this framework does not imply that microbes directly monitor or interpret neural activity in a cognitive sense. Rather, microbial communities respond adaptively to host-derived physiological signals, including changes in diet, stress hormones, immune activity, autonomic output, and gut motility, while microbial metabolites and signalling molecules influence neural and behavioural states through immune, endocrine, metabolic, and vagal pathways. The ‘closed-loop’ therefore refers to dynamic system-level coupling between microbiota, host physiology, and neural processes across multiple timescales. Recognising the microbiota as a functional layer of cognition within this distributed framework not only reframes our understanding of thought and behaviour but also opens new avenues for exploring cognitive disorders, self-regulation, and the boundaries of the mind.

## Positioning the microbiota within cognitive theory

This framework is situated within existing theories of cognition, including embodied cognition, extended mind theory, and predictive processing 1, 2, to clarify how microbial processes can be understood as part of a broader cognitive system. The idea that **gut microbiota** forms a functional layer of cognition connects with, but also extends beyond, several established theories in cognitive science. Traditional brain-centred models assume that cognition occurs solely within neural tissue, treating bodily systems as secondary influences. In this opinion article, cognition is conceptualised as distributed information processing emerging from interactions across neural, bodily, and microbial systems 3, 4.

From this perspective, the microbiota is not merely a background factor influencing mood or physiology but an active contributor to the formation and regulation of cognitive states 1, 2. The extended mind framework suggests that external tools, such as notebooks or smartphones, can become part of our cognitive system when they are consistently used to support thinking 1, 2. The microbiome differs in a crucial way: it is not a passive aid but a biologically active system that continuously senses changes in the host, adapts to them, and produces signals that influence motivation, emotional tone, and decision-making 1, 4.

Predictive processing models provide a helpful way to understand this interaction. These theories propose that the brain constantly generates predictions about the body and environment, updating these predictions based on incoming signals [3]. Microbial communities may contribute to this process by encoding patterns related to diet, stress, and environmental exposure, and by feeding this information back to the brain through metabolic and immune signalling channels 5, 6. In doing so, they may bias how the brain interprets internal states, assesses risk, or prioritises certain behaviours.

**Figure 1:**
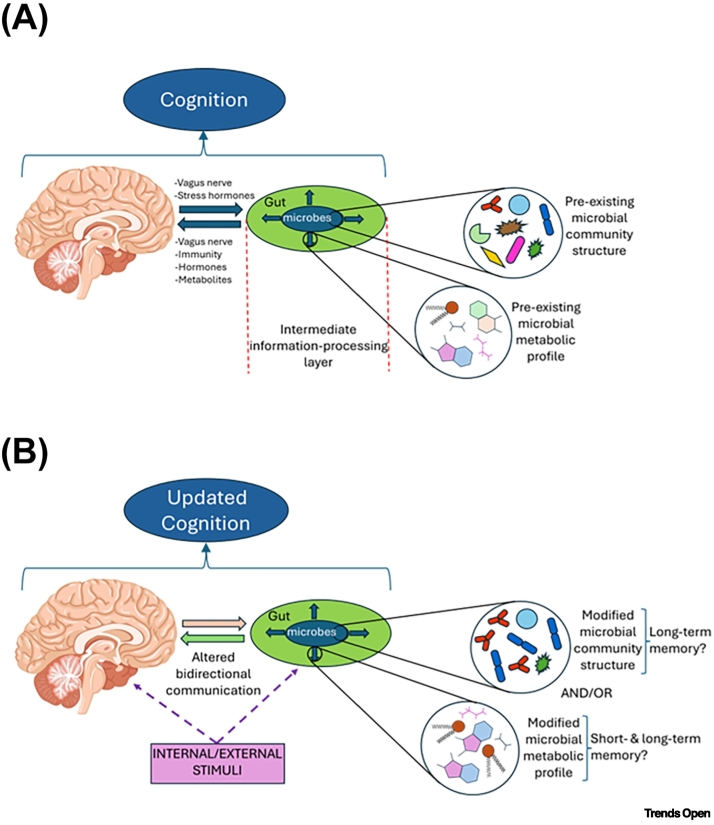
Key figure. The gut microbiota as an intermediate functional layer of cognition. (A) Under baseline conditions, cognition is shaped by bidirectional communication between the brain and the gut microbiota via neural, endocrine, immune, and metabolic pathways. Pre-existing microbial community structure and metabolic profiles influence gut–brain signalling and contribute to cognitive state. (B) Internal or external stimuli (e.g., stress, diet, learning, and environmental exposures) alter bidirectional brain–gut communication, leading to modifications in microbial community structure and/or microbial metabolic output. These microbiota-dependent changes feed back to the brain and are associated with updated cognitive states. Persistent alterations in microbial community composition and metabolic profiles are proposed to act as forms of biological memory, potentially contributing to short- and long-term cognitive processes. In this framework, the gut microbiota functions as an intermediate information-processing layer that integrates host- and environment-derived signals and modulates cognition over time.

Seen in this way, cognition emerges not only from neural circuits but also from coordinated activity across the brain, body, and microbial ecosystems, consistent with evidence that nonneural cellular networks can perform computation and contribute to information processing [4] (Figure 1, Key figure). The microbiota can be seen as an independent biological system that helps shape cognitive processes. This view keeps the science realistic while broadening what we consider part of a cognitive system.

## Microbial memory and cognitive modulation

Within this framework, memory is considered a component of cognition, referring specifically to the encoding, storage, and retrieval of information over time, whereas cognition more broadly encompasses processes such as perception, decision-making, and behavioural regulation. A useful reference is the classical model of memory comprising encoding, storage, and retrieval. In this context, encoding corresponds to environmentally or behaviourally induced shifts in microbial composition or function; storage is reflected in the persistence of these microbial states over time; and retrieval occurs when these states influence host neural activity or behaviour upon re-exposure to relevant internal or external cues. The examples described below can be interpreted within this framework, illustrating how microbial dynamics may fulfil analogous roles to memory processes in neural systems.

Emerging evidence from preclinical models and human studies suggests that the gut microbiota can act as a repository of experience, encoding memory-like functional states that influence host cognition 4, 7 (Box 1). For example, in preclinical studies, **faecal microbiota transplantation** from behaviourally trained or young donor mice into germ-free or aged recipients has been shown to improve learning, memory performance, and synaptic plasticity [8]. In humans, dietary and probiotic interventions have been associated with changes in microbial composition alongside alterations in cognitive or affective outcomes, although causal evidence remains limited 6, 9. Unlike transient signalling events, these microbial configurations can persist and respond dynamically to environmental or physiological changes, effectively serving as a form of persistent biological state representation. Exposure to stress, behavioural conditioning, and/or environmental changes can alter microbial composition, which in turn shapes neural activity and emotional or cognitive responses 10, 11, 12. Importantly, these memory-like functional states are not solely explained by differences between microbial species or by the metabolites they produce. Even within a single species, microbes can generate a range of functionally distinct outputs, including metabolites, signalling molecules, and polymers, that vary depending on environmental context, growth phase, and host interactions 9, 13. In this way, microbes translate prior experience into active functional responses that modulate host neural circuits and behaviour. These microbial responses occur within a host-dependent physiological context. Host genetics, developmental history, stress exposure, immune activity, endocrine signalling, diet, and prior behavioural experience can all influence microbial composition and functional outputs, shaping how microbiota-derived signals are generated, transmitted, and interpreted by the host. Thus, MGBA communication is not driven by microbial activity alone but emerges from continuous interactions between microbial and host biological states.

Box 1 **Microbial memory and cognitive modulation**

Microbial memory-like states persist over time and can be shaped by stress, environmental changes, or behavioural conditioning. Faecal microbiota transplantation studies show that experience-encoded microbial communities can enhance learning and memory in recipient hosts. Metabolites, SCFAs, neurotransmitter precursors, and immune-modulating molecules are likely mediators of these effects.

Takeaway: microbes translate past experiences into functional signals that influence host behaviour and cognition.

Alt-text: Box 1

Therefore, functional adaptation need not occur solely at the level of small-molecule metabolites or neurotransmitter precursors. Microbes can also modulate host signalling through higher-order functional outputs whose biological activity is encoded in their molecular architecture. A notable example is the production of **exopolysaccharides (EPS)**, high molecular weight polymers whose composition, branching, and charge are dynamically regulated in response to environmental conditions, growth state, and host-derived cues 14, 15. Although structurally complex, EPS function as active signalling molecules, that is, distinct EPS variants produced by the same microbial species have been shown to elicit different immune and epithelial responses in animal models, including differential activation of pattern recognition receptors, modulation of cytokine profiles, and maintenance of barrier integrity 16, 17, 18.

In humans, supplements containing EPS from *Bifidobacterium breve* were associated with increased gut microbial diversity and improved glycaemic indices in prediabetic adults compared with a placebo, indicating functional impacts on host physiology beyond taxonomy [19]. Moreover, *ex vivo* fermentation of human faecal microbiota with beverages supplemented with EPS from *Leuconostoc mesenteroides* shifted community composition towards beneficial taxa and increased **short-chain fatty acid (SCFA)** production, supporting functional modulation of the human gut ecosystem [20].

Given that EPS expression can persist over time yet remain adaptable to host inputs, these polymers provide a plausible substrate for **memory-like microbial states** that translate prior experience into ongoing functional signalling. In this way, microbial identity is not fixed at the species level but dynamically instantiated through functional outputs, enabling past experience to shape microbiota–host communication without requiring changes in community composition. This within-species signalling flexibility supports the view that microbial contributions to cognition operate through informational encoding rather than simple modulation, consistent with a distributed model of cognition across brain, body, and microbiota.

The microbiome is influenced not only by aversive environmental factors but also by natural ageing. One study reported significant changes in gut microbial diversity in a healthy aged population, which may contribute to cognitive decline. Supporting a causal role, faecal microbiota transplantation from young or task-experienced mice to aged or naïve recipients has been shown to enhance learning, memory, and synaptic plasticity, suggesting that the microbiota can carry functional ‘experience’ capable of modulating cognition [8]. While these effects are attributed to microbial transfer, other factors present in the faecal material, such as metabolites, SCFAs, neurotransmitter precursors, vitamins, bile acids, or immune-modulating molecules, may also contribute to the observed cognitive improvements. Notably, studies have demonstrated that faecal metabolite concentrations decline with age in mice, with 67% of identified features showing significant downregulation in older animals [21]. Additionally, metabolic differences between young and aged mice have been observed, including variations in bile acids and other metabolites [22].

A more concrete test of this framework could involve learning paradigms such as maze navigation tasks in rodents, which are well established for assessing memory acquisition and flexibility. In such experiments, task-trained donor mice harbour microbial communities and metabolite profiles shaped by experience. Transplantation into germ-free or naïve recipients has been shown to influence learning performance and synaptic plasticity in preclinical studies [8], although the specific contribution of microbial versus nonmicrobial components remains unresolved.

Within an encoding-storage-retrieval framework, these designs can be interpreted as testing whether experience-dependent microbial states (encoding and storage) can be transferred and subsequently influence behavioural outputs in recipients (retrieval). Therefore, a key gap is the lack of experiments that directly isolate microbial functional states from associated metabolites or host-derived factors, making it difficult to determine whether observed effects reflect true information encoding within microbial systems or indirect modulation of host physiology.

## Closed-loop feedback systems: MGBA synchrony

Closed-loop feedback refers to a system in which outputs influence subsequent inputs, creating a dynamic cycle of mutual regulation. This concept originates from control theory and systems neuroscience, where it is used to describe processes in which neural activity, behaviour, and sensory input are dynamically coupled across time. In cognitive neuroscience, closed-loop frameworks have been applied to understanding perception, decision-making, and adaptive behaviour, where actions shape incoming information that, in turn, updates internal states.

Within this framework, the MGBA is conceptualised as a closed-loop system comprising interacting microbial and host components operating across multiple levels. These include: (i) microbial communities and their functional outputs, (ii) gut physiology, including epithelial and enteroendocrine signalling, (iii) immune and inflammatory pathways, (iv) endocrine and metabolic signalling systems, (v) autonomic and vagal neural pathways, and (vi) central neural circuits underlying cognition and behaviour. Together, these components form a dynamically interacting network in which signals are exchanged and updated across multiple temporal scales.

Here, this concept is extended to the MGBA. Although the MGBA is widely recognised as bidirectional, this framework emphasises continuous, dynamically coupled feedback in which microbial activity influences host neural and physiological states, while host behaviour, diet, and internal conditions recursively reshape microbial composition and function over time. Unlike static or loosely coupled bidirectional models, a closed-loop perspective emphasises the timing, sensitivity, and mutual adaptation of these interactions, such that changes in one component directly influence subsequent system states. In this sense, microbiota–brain interactions can be understood as a biologically distributed closed-loop system operating across multiple timescales. A central prediction of this framework is that the MGBA functions as a dynamically coupled feedback system, co-regulating behaviour and cognitive processes through continuous feedback. Although the MGBA is described as dynamically coupled, interactions operate across multiple timescales rather than through uniform continuous signalling. Rapid communication is mediated by neural (vagal and enteric), endocrine, and immune pathways, as well as fast-acting microbial metabolites and host neurotransmitter systems. Intermediate processes include circulating microbial metabolites, hormonal signalling, and immune modulation, while slower processes involve shifts in microbial community structure, host gene expression, and long-term behavioural adaptation. In this sense, ‘continuous coupling’ refers to ongoing system-level interaction across overlapping temporal scales rather than instantaneous bidirectional signalling at every level of the system.

A potential objection to framing the gut microbiota as a functional layer of cognition concerns temporal mismatch: microbial population dynamics often operate more slowly than neural firing or behavioural responses, although microbial gene expression and functional responses can occur on much shorter timescales, in some cases within minutes of environmental or host-derived perturbations [23]. However, cognition itself is not confined to millisecond-scale processes. Cognitive states emerge from interactions across multiple timescales, including rapid neural signalling, intermediate neuromodulatory and immune processes, and slower mechanisms such as synaptic plasticity, hormonal regulation, and homeostatic set points. The microbiota primarily operates at these slower and intermediate timescales, shaping baseline affective tone, predictive priors, learning rates, and behavioural dispositions rather than moment-to-moment decisions. Together, these observations suggest that microbial contributions span a range of temporal scales, from rapid functional responses to longer-term shifts in community composition, enabling interactions with host processes across both fast and slow domains. In this framework, microbial contributions resemble state-setting and memory-like components of cognition, while also incorporating faster functional responses, collectively complementing rather than competing with fast neural computation.

Consistent with this framework, the gut microbiota and brain are dynamically coupled, co-regulating behaviour and cognitive processes. In this view, gut microbial changes dynamically influence neural activity, emotional reactivity, and decision-making, while host behaviour, diet, physiological state, stress exposure, immune activity, and genetic background continuously reshape microbial composition and function. Empirical studies of the human gut microbiome indicate that taxa can shift on timescales of days or even hours in response to diet or other host behaviours (e.g., onset times of ~0.4–1.5 days in a dietary glycan intervention [24]), and the day-to-day absolute abundance of many genera can vary up to 100-fold in individuals over 6 weeks [25]. The potential secretion of metabolites by microbes could enable even faster communication with the host. These dynamics are consistent with a model in which the gut microbiota forms part of a continuous closed-loop feedback system with the brain, wherein changes in host physiology or behaviour reshape microbial communities that, in turn, influence neural, emotional, and decision processes. Cognition, therefore, emerges from the dynamic interplay between these systems rather than being localised solely within the brain (Box 2).

Box 2 **Experimental architectures for microbiota–gut–brain coupling**

To study distributed cognition empirically, experimental designs use microbial signals as active inputs, creating closed-loop feedback systems. Microbiota-derived fluctuations can trigger task changes, affective challenges, or cognitive load shifts, allowing observation of real-time host-microbe interactions. Temporal sampling, defined thresholds, and data pipelines enable measurement of coupling stability, response latency, and interaction strength. Computational tools can translate these signals into predictive or perturbation-driven experiments.

Takeaway: these approaches provide a framework for testing how microbial activity directly shapes cognitive and emotional responses in real time.

Alt-text: Box 2

Experimental approaches to test this hypothesis require simultaneous, high-resolution monitoring of both the microbiome and brain activity. For instance, microbial composition and functional outputs could be tracked in real time via metabolomics or advanced stool biosensors. Emerging biosensor technologies include ingestible, battery-free devices capable of monitoring gut metabolites and transmitting data wirelessly [26], engineered probiotic biosensors that detect inflammatory or metabolic signals and report via acoustic signals [27], and aptamer- or electronic-based faecal biosensors that detect specific bacterial species and functional activity [28]. These tools enable dynamic, *in situ* assessment of microbiota states and functions. In parallel, neural activity can be recorded using electroencephalogram or functional magnetic resonance imaging during behavioural tasks such as decision-making or stress responses. Experimental manipulations, such as introducing microbial metabolites or dietary interventions midtask, would allow researchers to observe reciprocal, feedback-driven changes across the microbiota–brain axis. For example, recent studies integrating microbiome-related -omics with neuroimaging and behavioural assessments have begun to identify associations between microbial functional outputs and cognitive or emotional states [29], although these remain largely correlational. Embedding such approaches within closed-loop experimental designs, in which microbial signals dynamically inform task conditions or interventions, would allow causal testing of microbiota–brain interactions in real time. This represents a key step towards operationalising a distributed model of cognition in experimentally tractable systems.

This closed-loop framework reconceptualises cognition as emergent and distributed across the brain, gut, and microbiota, arising from their continuous, dynamic interactions. Although the MGBA is recognised as bidirectional and feedback-driven, traditional models often depict microbes as passive responders to diet, stress, or disease, with cognition still brain-centred [30]. In contrast, microbial communities actively shape cognitive states, influencing decision-making, emotional regulation, and adaptive behaviour across relevant time scales. Conceptually, this can be visualised as a continuous feedback cycle in which host and microbial states are iteratively and reciprocally updated over time. For example, dietary interventions have been shown to rapidly alter microbial composition and metabolite production within days, with high-resolution temporal profiling revealing consistent and cascading microbial responses to dietary glycans [24], illustrating how behavioural inputs dynamically reshape microbial outputs that can feed back to the host. Behaviour thus emerges from a reciprocally shaped network in which gut microbes are integral participants rather than mere modulators. This perspective establishes the theoretical foundation for moving beyond correlational synchrony towards experimental systems capable of testing real-time causal integration between microbiota and cognition. Together, these concepts provide the basis for experimentally testing microbiota–brain interactions as dynamically coupled systems rather than static associations. The following section outlines how such interactions could be operationalised in real-time experimental designs.

## Experimental architectures for real-time microbiota–brain coupling

To move from conceptual theory towards experimentally testable systems, the distributed cognition framework can be operationalised using closed-loop experimental architectures. Closed-loop designs utilise microbial fluctuations to dynamically adjust task conditions, allowing researchers to observe how changes in the microbiota influence cognitive or emotional states in real time. By treating microbial activity as an experimental input, researchers can examine how host behaviour and microbial dynamics adapt reciprocally, revealing the temporal structure and strength of microbiota–brain coupling.

These architectures require tightly synchronised temporal sampling, predefined response thresholds, and pipelines that convert microbial variability into controlled experimental interventions. Within-subject perturbation-recovery cycles allow quantification of coupling stability, responsivity, and lag, while metrics such as coherence, response latency, and coupling strength operationalise microbiota–brain interaction as a measurable variable.

Existing preclinical and clinical studies provide growing support for microbiota–cognition associations relevant to this framework. In animal models, microbiota manipulations, including germ-free conditions, faecal microbiota transplantation, and probiotic interventions, have been associated with alterations in learning, memory, emotional behaviour, and synaptic plasticity 7, 8, 11. In humans, microbiome-related omics integrated with neuroimaging and behavioural assessments have identified associations between microbial composition or functional pathways and cognitive performance, emotional processing, and altered brain network activity [29]. Although these studies remain largely correlational, they support the broader premise that microbial dynamics are linked to cognitive and neural states, motivating the need for experimentally tractable, closed-loop approaches capable of testing causality in real time.

Integrating computational tools can further enhance experimental tractability. For example, real-time data streams from microbial sensors can inform adaptive task algorithms [19], enabling predictive or perturbation-driven manipulations. Combining these approaches with metabolomics, neuroimaging, and behavioural readouts [29] creates a multi-scale platform for assessing the causal role of microbial dynamics in cognition.

By anchoring the theoretical shift from microbes as passive modulators to active participants within a concrete experimental framework, these architectures make it possible to probe real-time, causally meaningful interactions across the microbiota–brain axis. Collectively, these approaches provide a framework for linking microbial dynamics, neural activity, and behaviour across shared temporal scales, enabling experimental investigation of causal microbiota-gut–brain interactions.

## Causal modelling: microbes as components of distributed cognitive systems

Building on these experimental frameworks, computational and causal modelling approaches may help clarify how microbial signals influence host behavioural dynamics under conditions of uncertainty. A central prediction of the distributed cognition model is that certain microbial species or communities function as active participants in host decision-making, particularly under conditions of uncertainty. Although several microbiota–brain associations are supported by preclinical and clinical evidence, the interpretation of microbes as contributors to distributed decision-making remains a conceptual model that requires direct experimental validation. Rather than serving solely as sources of metabolites or modulators of neural circuits, microbes can be conceptualised as informational agents that contribute to computation-like processes shaping behaviour (Box 3). This perspective frames the microbiota as an integral component of a cognitive network, capable of influencing exploratory choices, risk assessment, and adaptive strategies.

Box 3 **Microbes as components of distributed cognitive systems**

Certain microbial species or communities may function as contributors within the gut–brain system by influencing host decision-making and adaptive behaviour. In this regard, ‘contribution’ refers to consistent, measurable effects on information processing and behavioural outcomes, without implying consciousness, intentionality, or independent cognitive agency. Computational tools such as system-level models can represent microbial–host interactions as coupled processes that modulate probabilistic choices, reward sensitivity, or exploratory behaviour, while controlled experimental manipulations in animal models combined with functional microbial profiling enable causal testing of these effects.

Takeaway: microbes can shape decision-making and adaptive behaviour by modulating information flow in the gut–brain system, without implying independent cognition.

Alt-text: Box 3

Experimental and modelling approaches can be used to test this hypothesis. Computationally, agent-based modelling or systems neuroscience frameworks can simulate microbial–host interactions as multi-agent networks, where each microbial population contributes information that influences host decision-making dynamics. Experimentally, specific microbial species or communities can be introduced into germ-free or microbiota-depleted mice, followed by behavioural testing using paradigms such as maze navigation or probabilistic reward tasks. For example, the faecal virome can be selectively isolated and transferred into mice to investigate its causal impact on behaviour [31] By comparing microbiome structure with known functional outputs, such as those producing dopamine precursors versus γ-aminobutyric acid modulators [32], microbial metabolic profiles can be mapped onto behavioural strategies.

This approach allows microbes to be treated as active cognitive agents, participating in the distributed processing of information that underlies decision-making, emphasising their potential to encode, transmit, and integrate information within the gut–brain network. By combining computational modelling with *in vivo* testing, this framework provides a rigorous path for uncovering causal links between microbial communities and host cognitive processes. Although still largely theoretical, these approaches provide a structured framework for testing whether microbial functional states contribute to behavioural adaptation in ways that extend beyond simple physiological modulation.

## Functional parallels between microbiota and brain networks

Viewed through the lens of distributed cognition, the gut microbiota may function in ways analogous to established neural systems. Just as excitatory and inhibitory networks balance information flow in the brain [33] microbial communities could modulate the timing, amplitude, and composition of signals reaching the central nervous system, influencing overall network dynamics. Similarly, baseline microbial activity may parallel the brain’s default mode network [34] maintaining a form of internal ‘readiness’ or homeostatic integration, while context-dependent shifts in microbial function resemble task-induced modulation of neural circuits, such as the fronto-parietal and salience networks [35]. In this framework, microbial signalling might also participate in the modulation of memory engrams, which are distributed neural ensembles that encode and retrieve experience [36], by influencing network excitability, synaptic plasticity, and communication between neural networks. Such interactions suggest that aspects of memory formation and retrieval may extend beyond traditional neural boundaries, reflecting a broader, biologically distributed substrate for cognition.

To fully understand the dynamic interactions between the gut microbiota and brain function, it is crucial to continue measuring microbial activity, including functional potential via metagenomics, metatranscriptomics, and/or metabolomics, alongside brain metrics. Several studies have begun to profile microbial function in various brain disorders, revealing associations between altered microbial pathways (e.g., neurotransmitter or SCFA production) and cognitive or emotional outcomes 37, 38. However, these remain observational studies, providing no direct evidence of cause and effect. To move beyond correlation, future research could integrate these microbial measures with the noninvasive techniques described above and extend to emerging technologies that track **vagus nerve** signalling and related microbiome–vagal pathways, including immune responses, in real time [39]. Combining functional microbiome profiling with direct measures of gut–brain communication could provide critical insight into how the microbiota actively shapes neural network dynamics and behaviour, opening the door to uncovering causal mechanisms.

## Critical challenges and conceptual boundaries

Despite the functional parallels described above, several conceptual and practical boundaries must be acknowledged. First, there is a risk of anthropomorphism, and attributing cognitive agency to microbes should be carefully distinguished from metaphorical language. Clear criteria are needed to differentiate microbial cognition—defined as the capacity to encode, process, and transmit information influencing host behaviour—from mere modulatory effects on neural circuits 1, 2, 5, 10, 30. The present framework does not propose that microbes possess intentionality or directly sense neural representations; rather, it conceptualises microbial communities as adaptive biological systems whose functional states participate in recursive host signalling loops that can shape cognitive processes indirectly. Second, the functional analogy to neural systems has inherent limits; while microbial communities can influence network dynamics, they lack the structured connectivity, excitatory-inhibitory balance, and synaptic plasticity characteristic of the brain 33, 34, 35. Third, measurement constraints remain substantial, including temporal resolution, specificity of microbial metabolite detection, and noninvasive monitoring of *in situ* microbial activity, all of which impose practical limits on experimental inference 23, 24, 25, 26, 27, 28. Finally, ethical considerations arise for interventions targeting microbial systems, especially in humans, where manipulation of the microbiota could have unforeseen effects on cognition, emotion, or behaviour [40]. Addressing these challenges is necessary to build a robust and well-grounded model of distributed cognition that recognises the role of microbes without exaggerating their similarity to brain networks.

## Towards a distributed model of cognition

Reframing cognition as a distributed microbiota–brain system offers a powerful conceptual shift with direct translational potential. By viewing the microbiome as an active component of cognition rather than just an influencing factor, we can create a framework that links molecular, neural, and behavioural processes in real time. This perspective suggests that microbial dynamics, through their capacity to encode experience, modulate signalling, and participate in feedback loops, may directly contribute to cognitive stability and flexibility.

In the context of cognitive and neuropsychiatric disorders, such as psychosis, mood disorders, and dementia, this perspective opens new therapeutic avenues. This framework may be particularly beneficial in addressing treatment-resistant forms of psychosis and depression, where conventional neurotransmitter-targeted interventions often fail to achieve sustained symptom relief. That is, interventions that restore or reprogram microbial function, such as targeted probiotics, dietary modulation, or microbial metabolite mimetics, could recalibrate dysregulated neural network dynamics or excitatory/inhibitory imbalances.

Although adjunctive probiotic and prebiotic interventions are already being investigated in brain disorders, reframing these approaches within a model of microbial-neural synchrony emphasises that their potential benefits may go beyond simply altering neurotransmitter levels. Instead, such treatments might help restore coordination across neural networks, improving the dynamic balance and stability of brain activity. By highlighting this network-level perspective, we can better understand how microbial interventions could recalibrate cognitive and emotional processes in treatment-resistant conditions.

Emerging AI and computational tools will be critical for implementing this distributed cognition framework. Machine learning and causal inference models represent the next step beyond current association studies of brain disorders and microbiome alterations. By integrating multi-scale data, including metagenomics, metabolomics, neuroimaging, and behavioural readouts, these models can move beyond merely observing correlations to identifying predictive signatures and potential causal mechanisms underlying cognitive states or disorder subtypes. Recent studies provide strong support for this integrative approach as the way forward in the field 29, 41. Agent-based and multi-agent reinforcement learning models could simulate microbiota–brain interactions, helping to uncover emergent properties such as microbial contributions to decision-making under uncertainty or adaptive flexibility. These approaches would not only enhance mechanistic understanding but could guide personalised, microbiome-informed interventions for cognitive health.

Ultimately, embracing cognition as a networked property of the host and microbiota challenges traditional boundaries of neuroscience and psychology. It suggests that thought, memory, and decision-making emerge from the synergy of multiple living systems, each contributing to the flow of information that defines the mind. Integrating microbial function into cognitive science is not simply an expansion of scope; it is a step towards a truly biological theory of cognition, one that reflects the deeply interdependent dynamics between the brain, body, and microbiome.

## Concluding remarks and future perspectives

Viewing cognition as a networked property of the brain, body, and microbiota reframes microbes as active contributors rather than passive modulators. Memory, decision-making, and emotional regulation emerge from interactions across this dynamic system.

Future work should test causal links using closed-loop feedback experiments, longitudinal microbiome manipulations, and real-time neuroimaging combined with metabolomics. Computational models can guide these studies and reveal emergent properties of microbiota-gut–brain interactions.

Therapeutically, targeted probiotics, dietary interventions, or microbial metabolite mimetics could help restore neural network balance in cognitive or psychiatric disorders. Integrating experimental and computational approaches will clarify the mechanisms, timing, and functional impact of microbial contributions, advancing a biologically grounded theory of distributed cognition (see Outstanding questions).

Outstanding questions How can we define clear criteria to distinguish microbial contributions to cognition from mere modulatory effects on the brain?

Which microbial species or communities exert the strongest causal influence on specific cognitive processes, and under what conditions?

What are the temporal dynamics and latency of microbiota-gut-brain feedback loops during behaviour and decision-making?

How can closed-loop experimental systems be optimised to manipulate microbial signals and measure real-time cognitive outcomes in humans and animal models?

To what extent do microbial memory-like states persist, adapt, and generalise across different environmental or experiential contexts?

How do interactions between microbiota, host metabolism, and neural networks integrate to support distributed cognition?

Can computational models reliably predict microbiota contributions to cognition, and how can these predictions be validated experimentally?

What ethical considerations arise from manipulating microbial communities to influence cognition in research or clinical interventions?

How do ageing, disease, or environmental perturbations alter the capacity of the microbiota to function as a semiautonomous cognitive layer?

Could integrating multi-omics, neuroimaging, and behavioural data uncover previously unrecognised mechanisms of microbiota–gut–brain co-regulation?

Alt-text: Outstanding questions

Key references

1. Boem, M. *et al.* (2022) Out of our skull, in our skin: the microbiota-gut-brain axis and the extended cognition thesis. Biol. Philos. 37, 45. 10.1007/s10539-021-09790-6

This article establishes a conceptual foundation for viewing microbiota–gut–brain interactions within an extended cognition framework. It supports the idea that microbial processes may contribute to cognition, a premise expanded here into a distributed and testable model.

2. Constant, A. *et al. (2022)* Extended active inference: constructing predictive cognition beyond skulls. Mind Lang. 37, 373–394. 10.1111/mila.12330

This paper argues that cognition can extend beyond the brain and emerge from interactions among connected biological systems. It provides a theoretical foundation for the idea that microbes, body physiology, and neural processes may work together to influence cognitive states.

3. Fields, C. and Levin, M. (2024) Sensing, feeling, and the origins of cognition: life as a continuum of basal cognitive systems. Systems 12, 160

This work argues that information processing and adaptive behaviour are not limited to nervous systems but occur across living systems. It provides a theoretical basis for considering how microbial adaptive responses may contribute to cognition beyond the brain.

4. Zorgani, A. and Das, B.C. (2024) Exploring the memory of the gut microbiome: a multifaceted perspective. Front. Microbiomes 3, 1363961. 10.3389/frmbi.2024.1363961

This paper reviews evidence that microbial communities can retain history-dependent functional states and respond differently based on prior experiences. It provides an important framework for understanding microbial memory-like processes and their potential influence on host biology.

5. Cerna, C. *et al.* (2024) Fecal microbiota transplantation from young-trained donors improves cognitive function in old mice through modulation of the gut-brain axis. Aging Dis. 16, 3649–3670. 10.14336/AD.2024.1089

This study provides important experimental evidence that microbiota from behaviourally experienced donors can improve learning and memory outcomes in recipients. It supports the idea that experience-dependent microbial states may influence cognitive function.

Alt-text: Key references

## Author contributions

Conceptualisation: P.W.J.B. developed the central conceptual framework, integrated perspectives from microbiome research and cognitive theory, and defined the scope and structure of the manuscript.

Writing original draft: P.W.J.B. prepared the original manuscript draft, including the development of the main conceptual arguments and the review of the relevant literature.

Review and editing: P.W.J.B. revised the manuscript for intellectual content, clarity, and structure and addressed reviewer comments while preparing the final version.

## Glossary

**Exopolysaccharides (EPS)**: high-molecular-weight sugar polymers secreted by gut microbes. They can modulate host immune responses, epithelial integrity, and microbial community composition. EPS expression changes in response to environmental or host-derived cues, supporting memory-like microbial states.
**Faecal microbiota transplantation**: the transfer of gut microbial communities from a donor to a recipient. Experimentally, faecal microbiota transplantation can test whether experience-encoded microbial states or metabolites influence cognition, learning, or behaviour in recipients.
**Gut microbiota**: the diverse community of microorganisms, including bacteria, fungi, archaea, and viruses, living in the gastrointestinal tract. They influence host physiology, immune function, metabolism, and cognitive processes.
**Memory-like microbial states**: persistent, history-dependent functional configurations of microbial communities shaped by prior environmental, dietary, or host-related exposures. These states reflect stable or semistable shifts in microbial composition and function that can influence host physiology and behaviour via metabolites, immune signalling, and other bioactive outputs.
**Microbiota–gut–brain axis (MGBA)**: the bidirectional communication network linking the gut microbiota and the brain via neural, immune, endocrine, and metabolic pathways. This axis allows microbial communities to influence brain function and behaviour, and for brain states to shape gut microbial composition and activity.
**Short-chain fatty acids (SCFAs)**: metabolic products of microbial fermentation of dietary fibres, primarily acetic, butyric, lactic, and propionic acids. SCFAs can modulate host metabolism, immune responses, and brain function, influencing behaviour and cognitive states.
**Vagus nerve**: a cranial nerve that connects the gut and brain, transmitting sensory information from the gut to the central nervous system. It can mediate microbial effects on cognition, emotion, and behaviour.

## References

[1] Boem M. (2022). Out of our skull, in our skin: the microbiota–gut–brain axis and the extended cognition thesis. Biol. Philos..

[2] Boem M. (2023). Minding the gut: extending embodied cognition to the gut complex. Philos. Psychol..

[3] Constant A. (2022). Extended active inference: constructing predictive cognition beyond skulls. Mind Lang..

[4] Fields C., Levin M. (2024). Sensing, feeling, and the origins of cognition: life as a continuum of basal cognitive systems. Systems.

[5] Zorgani A., Das B.C. (2024). Exploring the memory of the gut microbiome: a multifaceted perspective. Front. Microbiomes.

[6] Białoń N. (2024). Gut-brain axis and neuroplasticity in health and disease: a systematic review. AIMS Neurosci..

[7] Alavian F., Safaeian M. (2025). How the gut microbiome shapes learning and memory: a comprehensive review. IBRO Neurosci. Rep..

[8] Cerna C. (2024). Fecal microbiota transplantation from young-trained donors improves cognitive function in old mice through modulation of the gut-brain axis. Aging Dis..

[9] Sarkar A. (2016). Psychobiotics and the manipulation of bacteria-gut-brain signals. Trends Neurosci..

[10] Foster J.A. (2017). Stress & the gut-brain axis: regulation by the microbiome. Neurobiol. Stress.

[11] Li N. (2019). Fecal microbiota transplantation from chronic unpredictable mild stress mice donors affects anxiety-like and depression-like behavior in recipient mice via the gut microbiota-inflammation-brain axis. Stress.

[12] Castells-Nobau A. (2026). Gut microbial modulation of 3-hydroxyanthranilic acid and dopaminergic signalling influences attention in obesity. Gut.

[13] Ntiri E.S., Chun Nin Wong A. (2025). Microbial metabolites as engines of behavioral variation across animals. Gut Microbes.

[14] Caggianiello G. (2016). Exopolysaccharides produced by lactic acid bacteria: from health-promoting benefits to stress tolerance mechanisms. Appl. Microbiol. Biotechnol..

[15] Ferrario C. (2016). Modulation of the eps-ome transcription of bifidobacteria through simulation of human intestinal environment. FEMS Microbiol. Ecol..

[16] Fanning S. (2012). Bifidobacterial surface-exopolysaccharide facilitates commensal-host interaction through immune modulation and pathogen protection. Proc. Natl. Acad. Sci. U. S. A..

[17] Hidalgo-Cantabrana C. (2016). Effect of a ropy exopolysaccharide-producing *Bifidobacterium animalis* subsp. *lactis* strain orally administered on DSS-induced colitis Mice model. Front. Microbiol..

[18] Salazar N. (2019). Functional effects of EPS-producing *Bifidobacterium* administration on energy metabolic alterations of diet-induced obese mice. Front. Microbiol..

[19] Beteri B. (2024). Impact of combined prebiotic galacto-oligosaccharides and *Bifidobacterium breve*-derived postbiotic on gut microbiota and HbA1c in prediabetic adults: a double-blind, randomized, placebo-controlled study. Nutrients.

[20] Bisson G. (2025). Development of a bio-functional fermented soy beverage supplemented with microbial exopolysaccharides and its effect on the human gut microbiome *in vitro*. Food Funct..

[21] Best L. (2025). Metabolic modelling reveals the aging-associated decline of host-microbiome metabolic interactions in mice. Nat. Microbiol..

[22] Binyamin D. (2025). The microbiome is associated with obesity-related metabolome signature in the process of aging. NPJ Biofilms Microbiomes.

[23] Lacoux C. (2020). Dynamic insights on transcription initiation and RNA processing during bacterial adaptation. RNA.

[24] Creswell R. (2020). High-resolution temporal profiling of the human gut microbiome reveals consistent and cascading alterations in response to dietary glycans. Genome Med..

[25] Vandeputte D. (2021). Temporal variability in quantitative human gut microbiome profiles and implications for clinical research. Nat. Commun..

[26] De La Paz E. (2022). A self-powered ingestible wireless biosensing system for real-time in situ monitoring of gastrointestinal tract metabolites. Nat. Commun..

[27] Buss M.T. (2025). Probiotic acoustic biosensors for noninvasive imaging of gut inflammation. Nat. Commun..

[28] Moreira M.J. (2024). Are aptamer-based biosensors the future of the detection of the human gut microbiome?—A systematic review and meta-analysis. Biosensors (Basel).

[29] Sheng C. (2023). An integrated neuroimaging-omics approach for the gut-brain communication pathways in Alzheimer’s disease. Front. Aging Neurosci..

[30] Costa C.F.F.A. (2024). Importance of good hosting: reviewing the bi-directionality of the microbiome-gut-brain-axis. Front. Neurosci..

[31] Ritz N.L. (2024). The gut virome is associated with stress-induced changes in behaviour and immune responses in mice. Nat. Microbiol..

[32] Chen Y. (2021). Regulation of neurotransmitters by the gut microbiota and effects on cognition in neurological disorders. Nutrients.

[33] Barzon G. (2025). Excitation-inhibition balance controls information encoding in neural populations. Phys. Rev. Lett..

[34] Buckner R.L. (2008). The brain’s default network: anatomy, function, and relevance to disease. Ann. N. Y. Acad. Sci..

[35] Schimmelpfennig J. (2023). The role of the salience network in cognitive and affective deficits. Front. Hum. Neurosci..

[36] Tonegawa S. (2018). The role of engram cells in the systems consolidation of memory. Nat. Rev. Neurosci..

[37] Cheng J. (2024). Gut microbiota-derived short-chain fatty acids and depression: deep insight into biological mechanisms and potential applications. Gen. Psychiatry.

[38] Mhanna A. (2024). The correlation between gut microbiota and both neurotransmitters and mental disorders: a narrative review. Medicine (Baltimore).

[39] Bu Y. (2024). Non-invasive ventral cervical magnetoneurography as a proxy of in vivo lipopolysaccharide-induced inflammation. Commun. Biol..

[40] Tegegne H.A., Savidge T.C. (2025). Gut microbiome metagenomics in clinical practice: bridging the gap between research and precision medicine. Gut Microbes.

[41] Wu Y., Xie L. (2025). AI-driven multi-omics integration for multi-scale predictive modeling of genotype-environment-phenotype relationships. Comput. Struct. Biotechnol. J..

